# Endocannabinoid System and Its Regulation by Polyunsaturated Fatty Acids and Full Spectrum Hemp Oils

**DOI:** 10.3390/ijms22115479

**Published:** 2021-05-22

**Authors:** Slavko Komarnytsky, Thirumurugan Rathinasabapathy, Charles Wagner, Brandon Metzger, Carolina Carlisle, Chinmayee Panda, Sara Le Brun-Blashka, John P. Troup, Saradhadevi Varadharaj

**Affiliations:** 1 Plants for Human Health Institute, North Carolina Research Campus, North Carolina State University, 600 Laureate Way, Kannapolis, NC 28081, USA; trathin@ncsu.edu (T.R.); cswagner@ncsu.edu (C.W.); 2 Department of Food, Bioprocessing & Nutrition Sciences, North Carolina State University, 400 Dan Allen Drive, Raleigh, NC 27695, USA; 3 Department of Plant and Microbial Biology, North Carolina State University, 100 Derieux Place, Raleigh, NC 27695, USA; 4 Standard Process Inc., Nutrition Innovation Center, 150 N Research Campus Drive, Kannapolis, NC 28081, USA; BMetzger@Standardprocess.com (B.M.); ccarlisle@Standardprocess.com (C.C.); cpanda@Standardprocess.com (C.P.); slebrunblashka@Standardprocess.com (S.L.B.-B.); jtroup@Standardprocess.com (J.P.T.); svaradharaj@Standardprocess.com (S.V.)

**Keywords:** ECS tone, phytocannabinoids, inflammation resolution, oxidative stress, systemic redox balance

## Abstract

The endocannabinoid system (ECS) consists of endogenous cannabinoids, their receptors, and metabolic enzymes that play a critical homeostatic role in modulating polyunsaturated omega fatty acid (PUFA) signaling to maintain a balanced inflammatory and redox state. Whole food-based diets and dietary interventions linked to PUFAs of animal (fish, calamari, krill) or plant (hemp, flax, walnut, algae) origin, as well as full-spectrum hemp oils, are increasingly used to support the ECS tone, promote healthy metabolism, improve risk factors associated with cardiovascular disorders, encourage brain health and emotional well-being, and ameliorate inflammation. While hemp cannabinoids of THC and CBD groups show distinct but complementary actions through a variety of cannabinoid (CB1 and CB2), adenosine (A2A), and vanilloid (TRPV1) receptors, they also modulate PUFA metabolism within a wide variety of specialized lipid mediators that promote or resolve inflammation and oxidative stress. Clinical evidence reviewed in this study links PUFAs and cannabinoids to changes in ECS tone, immune function, metabolic and oxidative stress adaptation, and overall maintenance of a well-balanced systemic function of the body. Understanding how the body coordinates signals from the exogenous and endogenous ECS modulators is critical for discerning the underlying molecular mechanisms of the ECS tone in healthy and disease states. Nutritional and lifestyle interventions represent promising approaches to address chronic metabolic and inflammatory disorders that may overlap in the population at risk. Further investigation and validation of dietary interventions that modulate the ECS are required in order to devise clinically successful second-generation management strategies.

## 1. Introduction

The endocannabinoid system (ECS) is a major signaling network comprised of endogenous, lipid-based, physiological ligands (endocannabinoids) that play a pro-homeostatic role in central and peripheral organs of the human body. It is believed to constitute a feedback loop for nutrient-energy metabolism, and regulates various cellular functions, immune responses, and stress adaptation pathways to support physiological homeostasis as human diets change due to evolutionary and environmental factors [1]. The physiological outcomes may range from simple functions like eating, sleeping, and relaxing, to the more complex, including neuroplasticity, metabolism, and inflammation [2]. The functional integration of the ECS system in the control of inflammation, energy metabolism, and emotional homeostasis has been directly connected to its ability to adequately perceive and respond to the paucity of these signals [3]. Chronic, excessive, or unresolved overstimulation of the ECS often results in systemic imbalance. This can trigger oxidative stress by increasing the levels of reactive oxygen species (ROS) that adversely affect protein oxidation [4], mitochondrial bioenergetics [5], cellular functions, and redox balance maintained in part by the nuclear factor erythroid 2-related factor 2 (Nrf2) [6,7].

**Figure 1:**
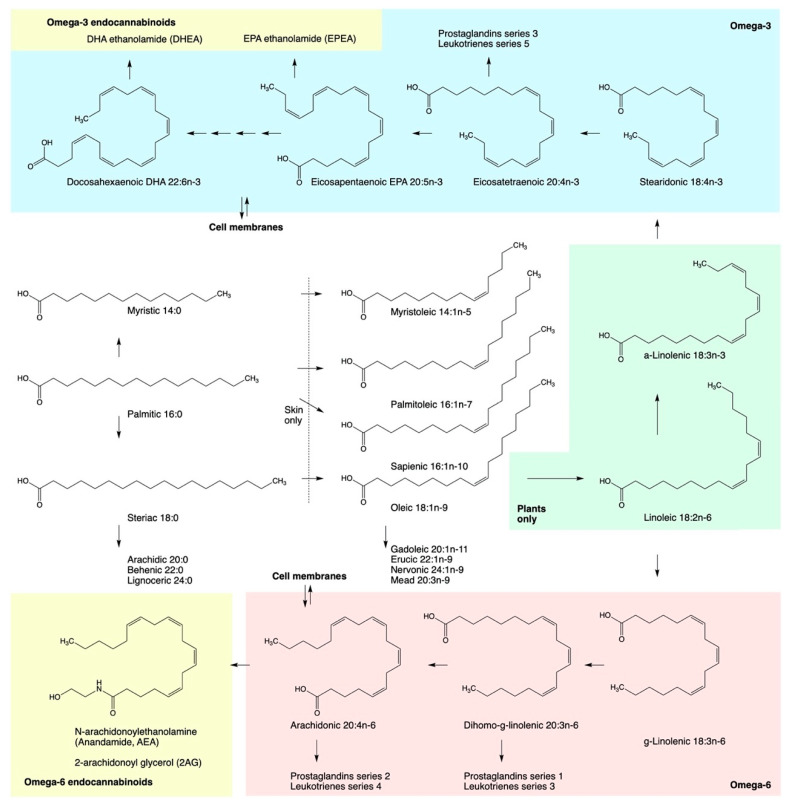
Pathways in the biosynthesis of polyunsaturated fatty acids including n-6 and n-3 essential fatty acids precursors, eicosanoid family metabolites, and endocannabinoids.

Endocannabinoids are metabolite products of dietary essential polyunsaturated fatty acids (PUFAs) (Figure 1). PUFAs are essential in terms of their critical role in maintaining the efficient structure and integral function of the cell membranes, and they must be obtained from the dietary sources [8]. Two essential dietary PUFAs, linoleic 18:2(n-6) and α-linolenic 18:3(n-3), serve as a starting metabolites for synthesis of omega-6 and omega-3 series fatty acids, albeit the metabolic pathway to the omega-3 eicosapentaenoic 20:5(n-3) (EPA) and docosahexaenoic 22:6(n-3) (DHA) acids in humans is very inefficient [9]. The omega-6 series fatty acid metabolites predominantly contribute to the synthesis of pro-inflammatory lipid mediators based on arachidonic acid 20:4(n-6), that also serves as a precursor for two major endogenous cannabinoid receptor ligands, anandamide (AEA) [10] and 2-arachidonoylglycerol (2-AG) [11]. On the other hand, the omega-3 series metabolites lead to formation of weakly anti-inflammatory groups of prostaglandins series 3 and leukotrienes series 5 [12], as well as less abundant classes of omega-3 derived endocannabinoids such as docosahexaenoylethanolamide (DHEA), eicosapentanoyl ethanolamide (EPEA), and related metabolites [13]. Both groups are further metabolized by the eicosanoid synthesizing enzymes: cyclooxygenase (COX), lipoxygenase (LOX) and cytochrome P450 epoxygenase (CYP450), to produce a variety of oxylipin metabolites including the specialized pro-resolving lipid mediators (SPM) that modulate the crosstalk between the ECS and immune systems of the body [14]. Dietary and nutritional supplementation strategies based on these metabolites also tend to ameliorate systemic metabolic and immune outcomes by resolving inflammation in adipose tissues and enhancing insulin sensitivity [15].

The selective affinity of other dietary lipid food components towards modulation of membrane-derived lipid modulator signaling is discussed in the subsequent sections below. Both naturally and dietary occurring lipid modulators and their oxidative metabolites interact with a much wider range of the ECS targets, including members of the cannabinoid receptors (CB), transient receptor potential channels (TRP), peroxisome proliferator-activated receptors (PPARs), and P450 enzymes [16]. This review therefore attempts to outline the progress and gaps in the field of nutrition and dietary interventions to support the ECS in managing inflammatory and oxidative challenges.

## 2. ECS Tone and Its Health Implications

The ECS is widely conserved among the vertebrate organisms [17]. Endogenous lipid messengers that mediate ECS signaling are synthesized on demand from the membrane phospholipid-derived arachidonoyl metabolites and can freely diffuse through cellular membranes due to their highly lipophilic nature. They include two well-studied endocannabinoids, the N-arachidonoylethanolamine (anandamide, AEA) and 2-arachidonoylglycerol (2-AG) [11], as well as a series of related molecules such as 2-arachidonylglycerylether (noladin ether, 2-AGE), O-arachidonoylethanolamine (virhodamine), and N-arachidonoyldopamine (NADA) [18]. Endocannabinoids activate presynaptic and astrocytic CB1 receptors distributed primarily in the central nervous system and CB2 receptors found in highest densities on the peripheral immune and hematopoietic cells [19]. Additional sets of G-protein coupled receptors including TRPV1 [20], GPR18, GPR55, and GPR119 [21] have been also suggested to mediate CB1/2-independent signaling, although their relevance to the physiological effects of cannabinoid stimulation remains unclear [22]. An unidentified endothelial receptor for endocannabinoids also mediates NO-independent vasodilation and endothelial cell migration [23].

Tissue-specific enrichment and distribution of CB1/2 receptors are highly relevant to their function. Central CB1 receptors mediate physiological control of psychoactive outcomes, anxiety and emotional wellbeing, food intake, energy metabolism, and contributes to the development of obesity and related metabolic risk factors when endocannabinoid activity is increased [24]. Neuroprotective, antinociceptive, and stress-induced analgesic activity of endocannabinoids is also predominantly mediated by central CB1 receptors that, upon activation, decrease neuronal excitability and neurotransmitter release by reducing intracellular Ca^2+^ stores, and induce centrally-mediated hypothermia [25]. Acute peripheral nociception and neuropathic pain, however, are also reduced by activation of CB2 receptors at peripheral sites and inhibition of local inflammatory hyperalgesia [26]. Peripheral and microglial CB2 receptors are strongly upregulated by pro-inflammatory and stress-related stimuli, and are critical in modulation of anti-inflammatory and tissue-protective activity by endocannabinoids [27]. At the molecular level, targeting CB2 leads to profound decreases in immune cell activation, inflammatory cytokine production, and inflammatory cell migration, generally in the absence of psychoactive effects. In immunocompromised subjects, however, CB2 driven outcomes can be harmful as they may contribute to the pathogen-induced inflammation or certain cancers [28]. Taken together, these observations suggest an intervention strategy to improve metabolic and immune outcomes that stimulates CB2 receptor signaling with global and peripherally restricted CB1 receptor activity, as summarized recently [29]. The wide and heterogeneous distribution of CB1 receptors in the brain [30], and recent findings of CB2 receptors not only in microglia but also in neurons [31], at the same time suggests that strengthening of the ECS tone by mild activation of central CB1 and CB2 is closely related with an emotional (anxiety, depression, PTSD) wellbeing, while overstimulation of CB1 activity induces psychotic symptoms [32]. 

An alternative strategy to improve ECS tone is increasing tissue levels of endocannabinoids by inhibiting their cellular uptake and metabolism [33]. This approach was proven successful with a series of pharmacological inhibitors targeting two key enzymes of ECS metabolism-fatty acid amide hydrolase (FAAH) that degrades AEA [34], and monoacylglycerol lipase (MAGL) that degrades 2-AG [35]. However, when an irreversible FAAH inhibitor PF-04457845 was evaluated in patients with osteoarthritic knee pain as a part of the randomized, placebo-controlled, phase II clinical trial (4 mg q.d. versus 500 mg b.i.d. naproxen for 2 weeks), it reduced FAAH activity in white blood cells (>96%), increased ECS substrates in plasma (4–10 fold), but failed to show a measurable analgesic effect [36]. Raising the global levels of endocannabinoids, therefore, is likely not sufficient to address the complexity and pleiotropy of their physiological effects. As endocannabinoids share a common lipid precursor with eicosanoids and can be metabolized by a shared set of cyclooxygenases, lipooxygenases and cytochrome P450 enzymes, the physiological outcomes of altered endocannabinoid metabolism can be tissue-specific, as it was shown in the brain tissue where 2-AG serves as a major arachidonate precursor for neuro-inflammatory prostaglandins [37].

## 3. Crosstalk between Inflammatory and ECS Signaling Mediators

Biological effects of arachidonic acid that serves as a precursor for endocannabinoids, are largely attributed to its enzymatic conversion to prostaglandins and thromboxanes by membrane-bound cyclooxygenases (COX), leukotrienes by cytoplasmic lipoxygenases (LOX), and lipoxides by cytochrome P450 epoxygenases [38]. Prostaglandins derived from arachidonic acid serve as secondary messengers of hydrophilic bioactive molecules such as glucocorticoids, non-steroidal anti-inflammatory drugs (NSAIDs), and statins to regulate blood cell aggregation, dilation of blood vessels, and vascular permeability, resulting in increased tissue edema, hyperemia, and fever to maintain the propagation of an inflammatory process. Both COX and LOX enzymes metabolize eicosanoids and endocannabinoid substrates with a similar efficacy [39]. While the oxidized endocannabinoids do not activate classical CB1/2 receptors, their cellular targets and respective biological mechanisms of action remain to be elucidated.

Eicosanoids serve also as key signaling molecules for activation of apoptotic neutrophils that drive the first line of host defense against microbes and external irritation/sensitivity signals from the environment, and lead to exacerbated tissue injury and chronic inflammation when not resolved in a timely fashion [40]. Uncontrolled production of noxious agents released by the immune cells to defend from the biotic and abiotic challenges such as proinflammatory and inflammatory mediators, ROS, and certain enzymes could also be detrimental. The transition from arachidonate-derived eicosanoids that drive inflammation (prostaglandins and leukotrienes) to anti-inflammatory lipoxins (such as LXA_4_) is driven in part by prostaglandins E_2_ and D_2_ that regulate the transcription of enzymes involved in lipoxin biosynthesis that complete the inflammatory feedback loop signaling [41]. 

Omega-3 series of essential fatty acids serve as substrates for synthesis of ligands that reduce incidence of infection and inflammation. Inflamed human endothelial cells used both EPA and DHA to release lipid-derived intermediates that are metabolized by leukocytes or glial cells to bioactive series E (RvE1) and series D (RvD1, PD1) metabolites [42]. Ethanolamide esters of DHA and EPA are also metabolized by neutrophils and brain tissues to active products that interact with CB1/2R receptors [43]. Activation of CB2 receptors on the immune cells by these cannabinoid ligands generally leads to the inhibition of CD14/TLR4 (Tol-like Receptor-4) inflammatory signaling pathway that drives pro-inflammatory T-helper-1 (TH1) immune response by increased interleukins (IL-1b, IL-6, IL-8) and tumor necrosis factor -α (TNF-α) production in macrophages [38]. 

Additionally, two sets of prostaglandins derived from dihomo-γ-linolenic acid 20:3(n-6) and eicosatetraenoic acid 20:4(n-3) show the relative anti-inflammatory activities of a lesser magnitude and remain virtually unexplored. It is postulated that these pathways shift the prostaglandin cascade away from the series 2 products to suppress cytokine production by monocytes and decrease platelet aggregation [44]. Hemp oil contains on average 3–6% γ-linolenic acid which serves as a precursor to series 1 prostaglandins and 15–25% α-linolenic acid that is metabolized into series 3 prostaglandins [45], and thus hold potential for activation of these pathways.

Recent developments in lipidomic methodologies uncovered a large number of additional oxylipins derived from other PUFAs, including those derived from the omega-6 fatty acids, as well as precursor linoleic and α-linolenic acids [46]. Oxylipins derived from different pathways and substrates can have same or opposing biological activity, and much remains to be elucidated about their actions and contributions to the individual oxylipin profiles.

## 4. Direct Modulation of ECS by Fatty Acids

Dietary fatty acids play a direct role in generation of lipid mediators that modulate inflammation and oxidative stress. While humans are very effective at desaturating two major circulating fatty acids, palmitic acid 16:0 and stearic acid 18:0 to their palmitoleic acid 16:1(n-7) and oleic acid 18:1(n-9) monounsaturated counterparts, they lack the enzymes to introduce the second unsaturated bond to form polyunsaturated linoleic acid 18:2(n-6) (a dietary precursor of omega-6 fatty acids) and α-linolenic acid 18:3(n-3) (a dietary precursor of omega-3 fatty acids) in their bodies. Dietary α-linolenic acid can be slowly metabolized into the long-chain polyunsaturated omega-3 fatty acids EPA and DHA when ingested directly, albeit with a lower efficiency [9]. Some marine organisms, especially fatty fish and shellfish accumulate large quantities of algae-derived EPA and DHA and serve as an excellent dietary source of these fatty acids [14]. 

On the contrary, synthesis of the omega-6 series of long chain polyunsaturated fatty acids via γ-linolenic acid 18:3(n-6) and dihomo-γ-linolenic acid 20:3(n-6) intermediates is very efficient [47]. Therefore, the intake of linoleic acid significantly exceeds that of α-linolenic acid, the elongation and desaturation of α-linolenic acid is inhibited because both molecules compete for the same enzymes. In order to keep the natural balance between omega-3 and omega-6 fatty acid metabolism, their dietary ratio should be kept in the range of 1:1 to 1:4, while a current estimate places it into 1:8 to 1:20 ratio for modern highly processed diets [48], resulting in part from the excessive use of vegetable oils high in linoleic acid [49]. The systemic imbalance that generally occurs under these circumstances, may trigger undesired immune, metabolic, and neurologic responses in the body [50]. It has been shown that various lipid dietary components may support key resolution pathways to inflammation, energy balance and metabolism via the ECS [51,52,53,54,55]. Recent clinical evidence from elder postmenopausal women receiving a fish oil supplement (720 mg EPA, 480 mg DHA) for 6 months confirmed increases of circulating omega-3 acyl-ethanolamide and omega-3-derived oxylipins that led to phenotypical responses in gene targets of the ECS system [56].

Diet is the main source of the arachidonic acid, which is subsequently stored in the form of phospholipid components of the cellular membranes. The cellular concentration of arachidonic acid is estimated from 3 pmol/10^6^ resting leukocytes (1 μM on a per volume basis) to 100 nmol/10^6^ inactivated human platelets (5 mM), with up to 50 μM local concentrations expected prior to its release from the cell [57]. Dietary fat intake exerts direct effects on ECS by increasing CB1 expression in the adipose tissue [58] and circulating levels of endocannabinoids AEA and 2-AG in the blood of healthy and insulin-resistant subjects [59], indicating chronic increase in the ECS tone directed at the preferential adipose tissue energy storage. These effects can be counteracted with additional EPA/DHA supplementation that changes the baseline levels of lipid mediators and decreases arachidonic acid derivatives [60], while also reducing expression of cannabinoid receptors and the ECS enzyme activity [61].

Further to signaling as lipid mediators, dietary fatty acids also play a critical role in the regulation of energy metabolism of monocyte/macrophage- and lymphocyte-based immune responses. Following monocyte activation or antigen recognition by lymphocytes in a lymph node, naïve immune cells undergo massive differentiation and expansion into M1/2 and Th1/2/17 phenotypes, respectively, which is generally achieved by switching from the β-oxidation of fatty acids to predominant use of glycolysis in the activated immune cells [62]. Supplementation of SPMs or omega-3 FAs ameliorates metabolic syndrome by resolving inflammation in adipose tissues and enhancing insulin sensitivity [15].

## 5. Hemp Oils as ECS Metabolic Modulators

**Figure 2:**
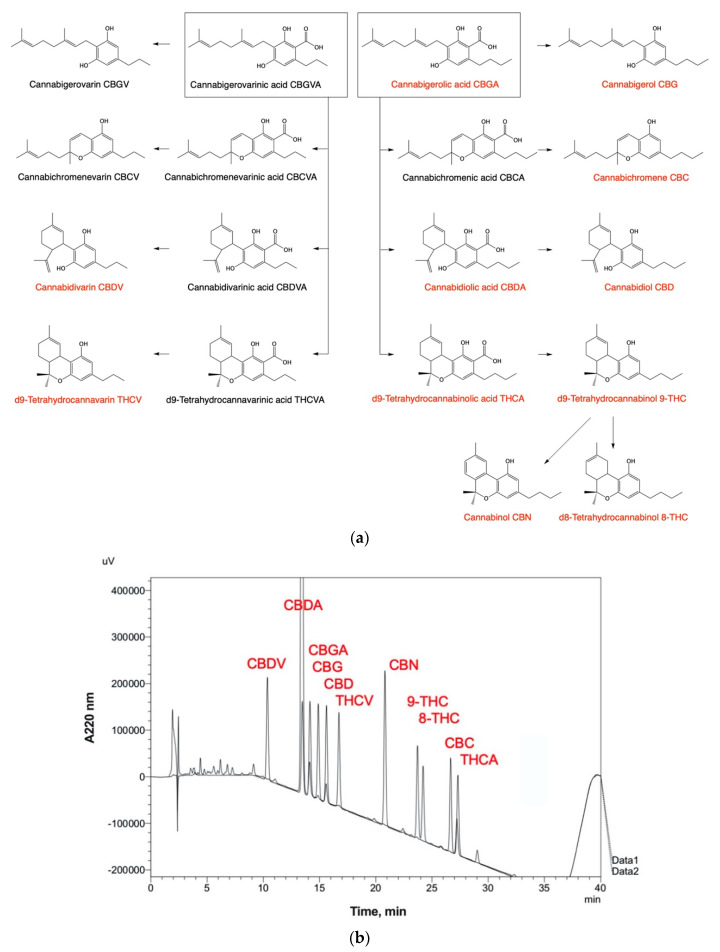
Major biosynthetic routes and cannabinoid metabolites found in *Cannabis sativa* tissues and botanical extracts. (**a**) The corresponding decarboxylation products are formed during collection, storage, and thermal processing of plant tissues. (**b**) HPLC-UV detection and full baseline resolution of 11 major cannabinoids using Shimadzu Prominence LC-2030C workflow as outlined in Table 1.

Hemp oils derived from the cannabis plant (*Cannabis sativa* L.) are a rich source of lipid bioactive compounds, including cannabinoids, β-caryophyllene, and polyunsaturated fatty acids [63] that potentially interact with the ECS. Following the first elucidation of structure and chemical synthesis of cannabinol (CBN), THC, and CBD in 1940s-60s [64] (Figure 2a), the first THC-binding site was described as CB1 receptor twenty years later [65], while specific molecular targets of CBD remained unknown until today. The therapeutic potential of cannabinoids, both as single bioactive agents and in combination with other bioactive plant compounds, to manage and alleviate multiple human health outcomes has received an excellent review [66]. Here we will briefly summarize key findings published since then, with a special emphasis on their ability to modulate the ECS tone. 

It is generally accepted that Δ^9^-THC found in *Cannabis* is a partial agonist of both CB1 and CB2 receptors similar to the endocannabinoids, with a slightly higher affinity for CB1 in the *Ki* range 4–40 nM [67], and exerts both psychotropic as well pain- and emesis-controlling effects in the CNS via CB1 and 5HT3 receptors activation [68]. Selective pharmacological inhibition of CB1 was achieved with a synthetic cannabinoid rimonabant (SR141716A, Acomplia) that reduced both food intake and body weight gain with a concomitant increase in psychiatric adverse events by modulating inhibitory neurotransmission in the central nervous system (CNS) [69]. Similar to endocannabinoids, Δ^9^-THC acts on neuronal presynaptic CB1 receptors to inhibit active neurotransmitter (i.e., gamma-aminobutyric acid) and stimulate dopamine release. Its high potency is likely responsible for a number of psychological side effects (anxiety, paranoia, panic and dysphoria) that occur more frequently with THC than with whole cannabis [70]. 

Δ^9^-THC affinity for both CB1 and CB2 exceeds that of other cannabinoids in the approximate potency order of Δ^9^-THC (4–40 nM) > Δ^8^-THC (35–40 nM) > THCV (60–125 nM) > CBN (100–1130 nM) > CBG (330–440 nM) > CBD (2860–4900 nM) [71]. Both Δ^8^-THC and CBN are degradation products of Δ^9^-THC found predominantly in heated, dried, and aged plant tissues, and act as antagonists of CB1/2 receptors at low doses and as agonists at high doses, possibly through competitive inhibition of endocannabinoid signaling or activity via an unrelated target [72]. Tetrahydrocannabivarin (THCV), a metabolic product of divarinolic rather than olivetolic acid, also antagonizes CB1R and acts as a partial agonist of CB2R. Together, these cannabinoids seem to act similar to Rimonabant in improving metabolic and glycemic control outcomes with greatly reduced psychotropic effects [73].

Cannabigerol (CBG), cannabichromene (CBC), and CBD is a group of non-psychotropic cannabinoids derived from the cannabinogerolic acid that show very low affinity for CB1/2 receptors, including a weak negative allosteric modulation of CB1 by CBD [74]. These cannabinoids modulate endocannabinoid tone indirectly by inhibiting anandamide uptake and reducing anandamide hydrolysis by fatty acid amide hydrolase (FAAH) and monoacylglycerol lipase (MAGL) [75].

Additionally, CBC shows potent agonist action at the TRPA1 channel [76], while CBD carries potent anti-inflammatory and immunosuppressive properties by activating adenosine A2A receptors [77] and attenuates anxiety, nausea, and depression by activation of 5HT1A receptors [78]. The vast variety of pharmacological effects associated with cannabinoids can therefore be partially explained by multiple interactions among different cannabinoids and their relative amounts found in cannabis interventions. The proportion of each cannabinoid class in the plant is strongly affected by growing conditions, geographical location, plant processing methods, and plant variety or chemotype. While the complexity of the described pharmacological interactions requires a widespread routine implementation of quality control and cannabinoid profile testing of hemp products, it also highlights the potential for the development of personalized interventions that modulate endocannabinoid tone and pathophysiological mechanisms of the ECS system. 

## 6. Phytochemical Complexity of Hemp Oils 

In 2017, the U.S. imported $67 million worth of hemp seed and fiber products, while the CBD market alone was worth $200 million, prompting nearly 60% of Kentucky hemp farmers to grow high CBD hemp varieties in 2018 [79]. Most farmers grow dioecious (separate male and female plants) high CBD hemp cultivars, and those plants show significant differences in cannabinoid profiles dependent on the target hemp tissue. Hemp accumulates cannabinoids primarily in the epidermal glandular trichomes in their native acid forms that decarboxylate to the respective neutral forms during storing and heat processing. Cannabinoids are present in decreasing amounts in flower bracts > flowers (female > male) > leaves (young > old) > stalks and branches > roots > seeds (hempseed oil) [80]. 

Overall, the hemp plant produces in excess of 140 cannabinoids that determine its potency and over 100 terpenes that define its distinctive flavor and aroma [81]. A typical high-resolution high-performance liquid chromatography (HPLC) analytical method with UV detection is capable of a full baseline resolution of 11 major cannabinoids including their native (acid) and neutral (decarboxylated) forms (Figure 2b). The HPLC analysis avoids conducting a previous decarboxylation of the samples and results in comprehensive identification and quantification of cannabinoids in their natural states. Quantitative determination of exact cannabinoid composition of hemp test samples and its derivatives, including extracts and oils, is critical because both cannabis type (variety, age, or target plant tissue such as flower buds, leaves, stems or roots) and agricultural practices (planting density, fertilization, light, humidity, harvest, and storage) dramatically influence the cannabinoid profile [82]. This inherent complexity of the cannabis phytochemical profile also allows for selective breeding strategies and blend reformulations towards the development of personalized interventions in response to individual patient outcomes. The modified analytical workflow for HPLC-UV quantitative determination of cannabinoids in cannabis tissues, oils [83], and biological samples are shown in Table 1.

Unique cannabinoid profiles of hemp crops sourced from different locations may determine their precise therapeutic and adverse side effects, further complicated by interactions between cannabinoids and terpenoids within the whole plant or full-spectrum hemp products responsible for the entourage effects [66]. Recent investigations summarized modulatory and therapeutic contributions of the cannabis terpenes [84] and flavonoids [85] to the entourage far beyond expectations considering their modest concentrations in the plant [86]. Notably, diverse compositional profiles of “full spectrum” (<0.3% THC) hemp oils that incorporate omega-3 PUFAs, cannabinoids, terpenes, and flavonoids in various ratios, are theorized to synergize through their biological activity, and the ECS system remains a primary target for such interactions [87]. Further industrial manipulations of crude hemp oils focus on selective elimination of THC below the detection limit (broad-spectrum oils), CBD distillates (refined to contain approx. 80% CBD with traces of other hemp phytoactives), and CBD isolates (crystallized CBD dissolved in hemp seed oil or another suitable oil carrier at approx. 99% CBD). Variations in the individual ECS tone and delivery formats (oral softgels, sublingual tinctures, or liposomal formulations) warrant further clinical research to substantiate the potential health promoting properties of different preparations.

## 7. ECS and the Oxidative Stress: Role of Dietary Antioxidants

The ECS tone is also critically associated with other systemic function of the body including inflammation and oxidative stress. Favorable redox and systemic homeostasis can be further promoted by incorporating bioactive botanicals that stimulate endogenous antioxidant networks signaling in an integrated fashion when the cannabinoids and terpenoids are delivered in appropriate quantities and ratios. Further, clinical evidence linking phytocannabinoids, endocannabinoids, and ECS suggests their unique potential role in the management of immune response and metabolic health outcomes for a well-balanced systemic function of the body [88].

Cellular defense mechanisms against oxidative, inflammatory, and toxic biochemical and environmental challenges constitute an important preservation mechanism to maintain optimal cellular structure and metabolism. The ability of the body to detoxify free radicals from an imbalanced physiological manifestation of ROS and reactive nitrogen species (RNS) may lead to a state of oxidative stress. The cellular redox homeostasis is challenged due to the formation of ROS and the prolonged oxidative stress is the underlying cause for unresolved inflammation leading to the development of several chronic metabolic conditions [89]. The uncontrolled ROS production and activity of the native antioxidant enzymes superoxide dismutase, glutathione peroxidase, nitric oxide synthase and catalase leads to increased peroxidation of polyunsaturated fatty acids, DNA oxidation, and cell death [90], therefore, the balanced redox system is critical in preserving the healthy metabolic conditions. Phytocannabinoids, including CBD, decreased the neuronal damage associate with β-amyloid accumulation and NADPH oxidase activity in SH-SY5Y neuroblastoma and BV-2 microglia cells, thus ameliorating oxidative stress [91]. Endogenous endocannabinoids like anandamide were also reported to attenuate inflammation and neurotoxicity in response to oxidative stress in PC12 cells [92], suggesting a significant cross-talk between the ECS and ROS signaling systems. Additionally, a 1:1 mixture of CBD and THC increased the ratio of reduced/oxidized glutathione and promoted autophagy in the brain of the tauopathic mice [93]. Further evidence to support this conclusion was obtained in a preclinical model of hepatic injury, where MAGL inhibition led to increased ECS tone and decreased oxidative stress and associated inflammation [35], while the modulation of distinct cannabinoid receptors promoted different cellular redox outcomes, with CB2 showing a protective response against ROS in isolated human macrophages [94]. In turn, activation of NADPH oxidase led to the upregulation of cannabinoid receptor expression in a feedback signaling loop to ameliorate oxidative damage in Schistosoma-infected mice [95]. CB2R receptor activation was also shown to activate Nrf-2 and induce heme oxygenase-1, a key cellular antioxidant in a mouse myocardial infarction model [96]. While Δ^9^-THC is typically oxidated one order of magnitude faster than other non-psychotropic cannabinoids, both CBD and CBG were shown to maintain redox stability and offer good UVA and UVB photoprotective properties [97].

Lipid peroxidation is a key feature of oxidative stress and associated pathogenesis of chronic metabolic and inflammatory diseases, in part by increased production of reactive aldehydes from the omega-6 and omega-3 PUFAs. The Nrf2 oxidation pathway is tightly connected to lipid peroxidation and the regulation of mitochondrial metabolism and function [98]. Additional dietary Nrf2 activators such as glucosinolates found in cruciferous vegetables may potentiate induction of genes that regulate defenses against oxidative stress, inflammation, and DNA-damaging electrophiles when consumed as a part of the healthy diet or dietary intervention strategy. Studies have shown that many isothiocyanates, particularly sulforaphane, increased expression of antioxidant enzymes via the activation of Nrf-2 dependent pathway [99]. The biological actions of glucosinolate-derived isothiocyanates (ITCs) with active myrosinase component enhanced the bioavailability of ITCs thereby enhancing Nfr2 activation and promoting cellular antioxidant capacity [100].

## 8. Other Dietary Interventions That Target ECS

Additional nutritional and dietary supplementation options to modulate ECS signaling and improve overall systemic health outcomes have been described in the literature. Over 20 different alkylamides with a variable degree of unsaturation of the aliphatic chains were found predominantly in the roots of *Echinacea angustifolia* DC. and *E. purpurea* (L.) Moench [101]. Of those, A1 alkylamide showed the highest selective partial agonist affinity for CB2R with Ki value of 60 nM [102]. A series of related N-alkylamides from oxeye daisy (*Heliopsis helianthoides* (L.) Sweet) and macamides from maca (*Lepidium meyenii* Walp.) with selective CB1 receptor activity (Ki of 400 nM) [103]. A sesquiterpene component of many essential oils, β-caryophyllene showed selective full agonist affinity for CB2R with Ki value of 150 nM [104]. Among 6 kavalactones naturally present in the roots of kava shrub (*Piper methysticum* G. Forst.), yangonin showed selective agonist activity against CB1R with Ki value of 720 nM [105]. On the other hand, falcarinol, a polyene fatty alcohol natural present in carrot root and seed oil (*Daucus carota* L.) provides a unique opportunity to downregulate CB1R signaling by serving as selective non-reversive agonist of this receptor with Ki value of 600 nM [106]. 

The selective affinity of dietary food components towards either CB1R or CB2R receptors suggests that dietary and supplementary interventions could be developed to support and contribute to the complex human pharmacology of the ECS system in addition to classic hemp cannabinoids and endocannabinoids of the human body. Food components could exert cannabinomimetic effects at the level of the endocannabinoid degrading enzymes FAAH and MAGL by directly inhibiting their activity and therefore increasing the systemic endocannabinoid tone. For example, non-endocannabinoid N-acylethanolamines found in many plants were found inactive for cannabinoid receptors, but nevertheless effective as restorative modulating lipid precursors that modify chronic inflammation in various infective, metabolic, and autoimmune states [107]. Functional interactions between the endocannabinoid system and essential ion micronutrient ions such as zinc and magnesium may be additive as it was shown in the animal models of depression [108]. 

Targeting stress responses and adaptation to internal and external stimuli are multistep processes that involve multiple molecular-cellular network at different levels. Collectively, the metabolic regulation by botanical interventions at the cellular and systemic levels are likely to be associated with a multitude of targets and physiological responses. Therefore, to achieve a desired biological outcome these phytoactive components can be preferably assembled and function collectively in the form of dietary matrix with multitargeting effects.

## 9. Conclusions

Dietary PUFAs are major sources for the biosynthesis of endocannabinoids, both by contribution of omega-6 fatty acids to the arachidonic biosynthesis pathway and modulation of the ECS tone by dietary omega-3 EPA and DHA esterified to phospholipids. Understanding this intricate balance could hold potential to reduce over activation of central and circulating endocannabinoids observed in subjects with metabolic disorders [109], as well as modifying the onset and progression of many chronic metabolic and immune disorders associated with elevated inflammation and oxidative stress. The role of multiple cannabinoids in the integrated and often opposing control of these outcomes may be considered direct evidence in favor of developing novel strategies that deliver appropriate quantities and ratios of different metabolites found in full-spectrum hemp oils and provide a more discriminating means of eliciting a balanced and personalized response. While no single bioactive principle may balance these complex health outcomes, novel intervention strategies and selected nutrient supplementation regimes that specifically target the ECS, and metabolic health outcomes could support key mediators and inflammatory resolution pathways critical in maintaining a well-balanced systemic function of the body.

## Figures and Tables

**Table 1:** Analytical workflow for HPLC-UV quantitative determination of 11 major cannabinoids in cannabis, its derivative extracts, oils, formulations, and biological test fluids.

Step	Detailed Description	Comments
1a	Hemp tissues and powdered samples: Weigh 1 g of air-dried samples from sealed bags in triplicate Add 20 mL of methanol/chloroform (9:1, *v*:*v*) Agitate in an orbital shaker for 30 min at 200 rpm and 37 °C Sonicate in an ultrasonic bath for 30 min at 37 °C Centrifuge for 5 min at 3000 rpm and RT, and collect supernatant Repeat twice and combine supernatants into a single sample Evaporate to dryness and dissolve in 1 mL methanol Collect supernatant and filter through a 0.45 µm PTFE syringe filter	Whole plant complexity, minor cannabinoids of importance, and a variable terpenoid profile may all contribute to beneficial *entourage* effect of hemp
1b	Hemp oils and liquid formulations: Add 400 µL of isopropanol to a 2 mL Eppendorf tube Add 10 µL of liquid sample and completely dissolve Vortex the sample for 30 sec at RT Add 400 µL of methanol to the mixture Vortex the sample for 30 sec at RT Filter through a 0.45 µm PTFE syringe filter	Hemp oil density is 0.92 (used as a conversion factor to calculate volume to weight ratio)
1c	Biological fluids (urine or plasma): Add 1 mL urine (3 mL plasma) and 20 µL of DDT to a 20 mL glass vial Add 3 mL of 100 mM sodium acetate buffer (pH 4.8) and mix briefly Add 375 μL of β-glucuronidase in acetate buffer Vortex briefly and incubate at 37 °C for 16 h Add 15 mL of ice cold 1% formic acid in acetonitrile Vortex briefly and sonicate in an ultrasonic bath for 3 min at RT Centrifuge for 5 min at 10,000 rpm and RT, and collect supernatant Add 15 mL methanol to the pellet and vortex briefly Repeat once and combine supernatants into a single sample Evaporate to dryness and dissolve in 200 µL methanol	4,4- Dichlorodiphenyltrichloro ethane (DDT, 50 µg/mL) is used as an internal analytical standard; β-glucuronidase (Abalone)
2	Standard curves over a linear dynamic range of 0.5–100 μg/mL (ppm)	Shimadzu #220-91239-21
3	Instrument: Shimadzu Prominence LC-2030C UV Column: Restek Ultra C18 (250 mm × 4.6 mm, 5 μm dp) Guard column: Restek Ultra C18 Guard (10 mm × 2.1 mm, 5 μm dp) Mobile-phase A: 0.1% formic acid in water Mobile-phase B: 100% acetonitrile Flow rate: 1 mL/min; Column temperature: 30 °C Injection volume: 20 μL; Detection: 220 nm	Cannabinoid totals are calculated as the sum of the neutral form and the acid form multiplied by the conversion factors (0.877 for THCA and CBDA; 0.878 for CBGA)

## Abbreviations

2-AG	2-arachidonoylglycerol
AEA	N-arachidonoylethanolamine
ALA	α-linolenic acid
CBD	cannabidiol
CB	cannabinoid receptor
COX	cyclooxygenase
DHA	docosahexaenoic acid 22:6(n-3)
ECS	endocannabinoid system
EPA	eicosapentaenoic acid 20:5(n-3)
FA	fatty acid
FAAH	fatty acid amide hydrolase
HPLC	high-performance liquid chromatography
IL	interleukin
LOX	lipoxygenase
MAGL	monoacylglycerol lipase
NRF2	nuclear factor erythroid 2-related factor 2
PPAR	peroxisome proliferator-activated receptor
PUFA	polyunsaturated omega fatty acid
ROS	reactive oxygen species
RNS	reactive nitrogen species
THC	tetrahydrocannabinol
TNF-α	tumor necrosis factor-α
TRP	transient receptor potential channel s (PPARs)
SPM	specialized proresolving mediators
